# “An Impediment to Living Life”: Why and How Should We Measure Stiffness in Polymyalgia Rheumatica?

**DOI:** 10.1371/journal.pone.0126758

**Published:** 2015-05-08

**Authors:** Sarah Louise Mackie, Rodney Hughes, Margaret Walsh, John Day, Marion Newton, Colin Pease, John Kirwan, Marianne Morris

**Affiliations:** 1 Leeds Institute of Rheumatic and Musculoskeletal Medicine, University of Leeds, Leeds, United Kingdom; 2 NIHR-Leeds Musculoskeletal Biomedical Research Unit, Leeds, United Kingdom; 3 Department of Rheumatology, Ashford and St Peter’s NHS Foundation Trust, Chertsey, United Kingdom; 4 PMRGCAuk Surrey, Chertsey, United Kingdom; 5 Department of Rheumatology, Leeds Teaching Hospitals NHS Trust, Leeds, United Kingdom; 6 Department of Rheumatology, University of Bristol, Bristol, United Kingdom; 7 University of the West of England, Bristol, United Kingdom; The James Cook University Hospital, UNITED KINGDOM

## Abstract

**Objectives:**

To explore patients’ concepts of stiffness in polymyalgia rheumatica (PMR), and how they think stiffness should be measured.

**Methods:**

Eight focus groups were held at three centres involving 50 patients with current/previous PMR. Each group had at least one facilitator and one rapporteur making field notes. An interview schedule was used to stimulate discussion. Interviews were recorded, transcribed and analysed using an inductive thematic approach.

**Results:**

Major themes identified were: symptoms: pain, stiffness and fatigue; functional impact; impact on daily schedule; and approaches to measurement. The common subtheme for the experience of stiffness was “difficulty in moving”, and usually considered as distinct from the experience of pain, albeit with a variable overlap. Some participants felt stiffness was the “overwhelming” symptom, in that it prevented them carrying out “fundamental activities” and “generally living life”. Diurnal variation in stiffness was generally described in relation to the daily schedule but was not the same as stiffness severity. Some participants suggested measuring stiffness using a numeric rating scale or a Likert scale, while others felt that it was more relevant and straightforward to measure difficulty in performing everyday activities rather than about stiffness itself.

**Conclusions:**

A conceptual model of stiffness in PMR is presented where stiffness is an important part of the patient experience and impacts on their ability to live their lives. Stiffness is closely related to function and often regarded as interchangeable with pain. From the patients’ perspective, visual analogue scales measuring pain and stiffness were not the most useful method for reporting stiffness; participants preferred numerical rating scales, or assessments of function to reflect how stiffness impacts on their daily lives. Assessing function may be a pragmatic solution to difficulties in quantifying stiffness.

## Introduction

Polymyalgia rheumatica (PMR) is an inflammatory musculoskeletal disease that affects older people. Core clinical features for the diagnosis of PMR include bilateral shoulder and/or hip pain, morning stiffness, and abnormal inflammatory markers such as the erythrocyte sedimentation rate [1]. “Morning stiffness” is considered an important diagnostic clue to presence of inflammatory symptoms, to differentiate “inflammatory” disorders such as PMR and rheumatoid arthritis (RA) from “non-inflammatory” disorders such as osteoarthritis [2]. PMR is treated with glucocorticoids (steroids), often for several years [2,3], at the cost of a significant burden of risks and adverse effects in this age group.[1] Monitoring disease activity in treated patients is therefore important, and may require physicians to ask different questions than they would use for diagnosis. Patient-reported outcome measures that capture the impact of the disease on patients’ lives are required for a core outcome set [4] that will allow optimal treatment strategies to be developed.

Current clinical guidelines recommend that patients treated for PMR should be monitored on the basis of symptoms (proximal pain, fatigue and morning stiffness), since conventional inflammatory markers can be misleading in PMR [1]. Factor analysis of an observational dataset suggested that morning stiffness duration, pain and laboratory markers may reflect different dimensions of disease [5]. However, it is not clear whether measures of stiffness and pain in PMR appear to diverge because they represent two distinct experiences of PMR, or because stiffness and pain are part of the same patient experience but stiffness is more difficult to measure accurately; morning stiffness duration has been reported to have poor test-retest reliability in PMR [6].

In preliminary informal discussions with groups of patients with PMR [5,7], patients rarely mentioned “morning stiffness”; and to some patients, stiffness and pain seemed to be part of the same experience. This challenged our preconceptions that the patient experience of stiffness in PMR could map directly onto the physician’s concept of inflammatory symptoms or “morning stiffness”. Qualitative research can produce novel insights into the patient experience in inflammatory disease [8]; here, we chose a qualitative approach to explore the patients’ concepts of stiffness and identify potentially valid ways of assessing or measuring stiffness in patients with glucocorticoid-treated PMR.

Focus group methodology allows discussion between participants [9] and the identification of common and individual experiences while minimising the influence of the researchers on their discussion [9, 10].

## Patients and Methods

Ethical approval was received from NRES Committee London-City and East (Ref: 12/LO0120: Mr Raj Khullar at the South West REC Centre, Whitefriars, Level 3, Block B Lewins Mead, Bristol, BS1 2NT); and governance approval was received from hospital research and development committees at Ashford and St Peter’s Hospital NHS Trust, Leeds Teaching Hospitals NHS Trust, and University Hospitals Bristol NHS Trust. Patients with a rheumatologist-confirmed diagnosis of PMR who were receiving treatment either currently or within the previous three years were invited to participate in focus groups. Prior to data collection all participants were provided with an information sheet and written informed consent was provided in advance, in line with the Declaration of Helsinki.

At all stages of the project, from planning the protocol to analysis and writing the results, advice and input was received from co-authors MN and JD, patient research partners with personal experience of PMR in accordance with the European League Against Rheumatism (EULAR) recommendations for patient research involvement [11].

Participants (n = 50; 36 females) were purposively selected from secondary care (rheumatology) clinics at three geographically distinct UK NHS trusts aiming for diversity of age, gender and disease duration. (Table 1).

**Table 1:** Participants in focus groups at the three centres.

	Chertsey (n = 19)	Leeds (n = 19)	Bristol (n = 12)
Number of focus groups conducted	3	3	2
Females, n (%)	13 (68%)	14 (74%)	9 (75%)
Age, years, median (range)	66 (59–79)	76 (53–84)	77 (45–86)
Disease duration, months, median (range)	28 (6–72)	9 (1–51)	15.5 (2–30)
White, n (%)	19 (100%)	19 (100%)	12 (100%)

Each focus group had one facilitator managing the contributions of each group member, and one rapporteur who made field notes to aid transcription accuracy. A common interview schedule was used (Table 2). The schedule was structured to elicit patient’s understanding and experience of PMR stiffness, how it impacted upon them, associated symptoms, diurnal variation and effectiveness of corticosteroid treatment, before asking them how they might measure stiffness to reflect their individual experiences. However, broader discussion emerged as patients began asking questions pertinent to them. These were monitored to ensure the purpose of the interview was maintained.

**Table 2:** Pre-defined interview schedule, used for all eight focus groups.

Question number	Question content
1	Definition of stiffness in words
2	Grading of severity of stiffness
3	Why is stiffness important, and how is it different from pain?
4	Does stiffness vary through the day and night, and should this be accounted for in measurement?
5	How does treatment change stiffness reporting?
6 (Bristol only)	If your PMR was an animal, what would it be?

Focus group discussions were digitally recorded, transcribed verbatim, anonymised, and cross-checked by group facilitators. The two Bristol focus groups took place after initial examination of data from the Chertsey and Leeds focus groups, allowing an extra question to be added to further promote discussions: ‘*If your PMR was an animal what would it be*?’ This question has been used in previous research to help patients describe their inflammatory arthritis and how this impacted upon them [12; 13].

Six stages of inductive thematic analysis were performed [14]: familiarization with the data; generation of initial codes by direct annotation of transcripts; searching for themes; reviewing themes; defining themes; and naming themes. In addition the penultimate interpretation was discussed with the two patient partners to check their interpretation of the data before the themes and their interpretation were finalised [11, 14]. Utilising focus groups and a broad interview schedule, we adopted a social constructionist perspective [15].

Analysis was led by MM, an experienced qualitative researcher who had not been involved in data collection, with input from all co-authors. Firstly the transcripts were systematically re-read and compared (MM read all transcripts; SLM read the Leeds transcripts; MW read the Chertsey transcripts) and initial data interpretation was discussed. Secondly two researchers (MM, SLM) returned iteratively to the annotated transcripts from all three sites, checking the validity of the interpretation of the data by clarifying meanings and codes, and building these up into themes guided by several rounds of discussion. As themes and sub-themes became defined, a conceptual model was developed to illustrate the interrelationships of these themes. As the intention of this work was to inform the development of a core outcome set for PMR, the development of this model was informed by the World Health Organisation International Classification of Functioning, Disability and Health, which classifies outcome measures into those affecting body functions and structure, activity and participation.[16]

In this report, direct quotes are annotated with the centre (SPH: St Peter’s Hospital, Chertsey; L, Leeds; B, Bristol) followed by the focus group number, the gender of the speaker and the line number(s) of the relevant transcript.

## Results

To present these data, themes will be cited in italics and verbatim quotes identified by location/focus group/gender/line number. Guided by the interview schedule, *stiffness* was discussed in relationship to *pain*, but participants also identified *fatigue* as an important symptom in PMR. Stiffness tended to be discussed in terms of impact on their functional ability (*PMR affects the things we do*); and on how they structured their day because of the 24-hour (diurnal) variation in symptom severity (impact on daily schedule: *PMR always on your mind*). *Measurement of stiffness* was influenced by all of these themes and provided a complementary perspective on patients’ experience of stiffness in PMR. Themes were also identified relating to the *emotional and social impact* of the symptoms, and participants’ use of self-management strategies to minimise the disruption to their lives. While clearly important to these patients, this was felt to go beyond the original research question relating to measurement of stiffness itself, therefore these will be presented in a separate report.

**Fig 1:**
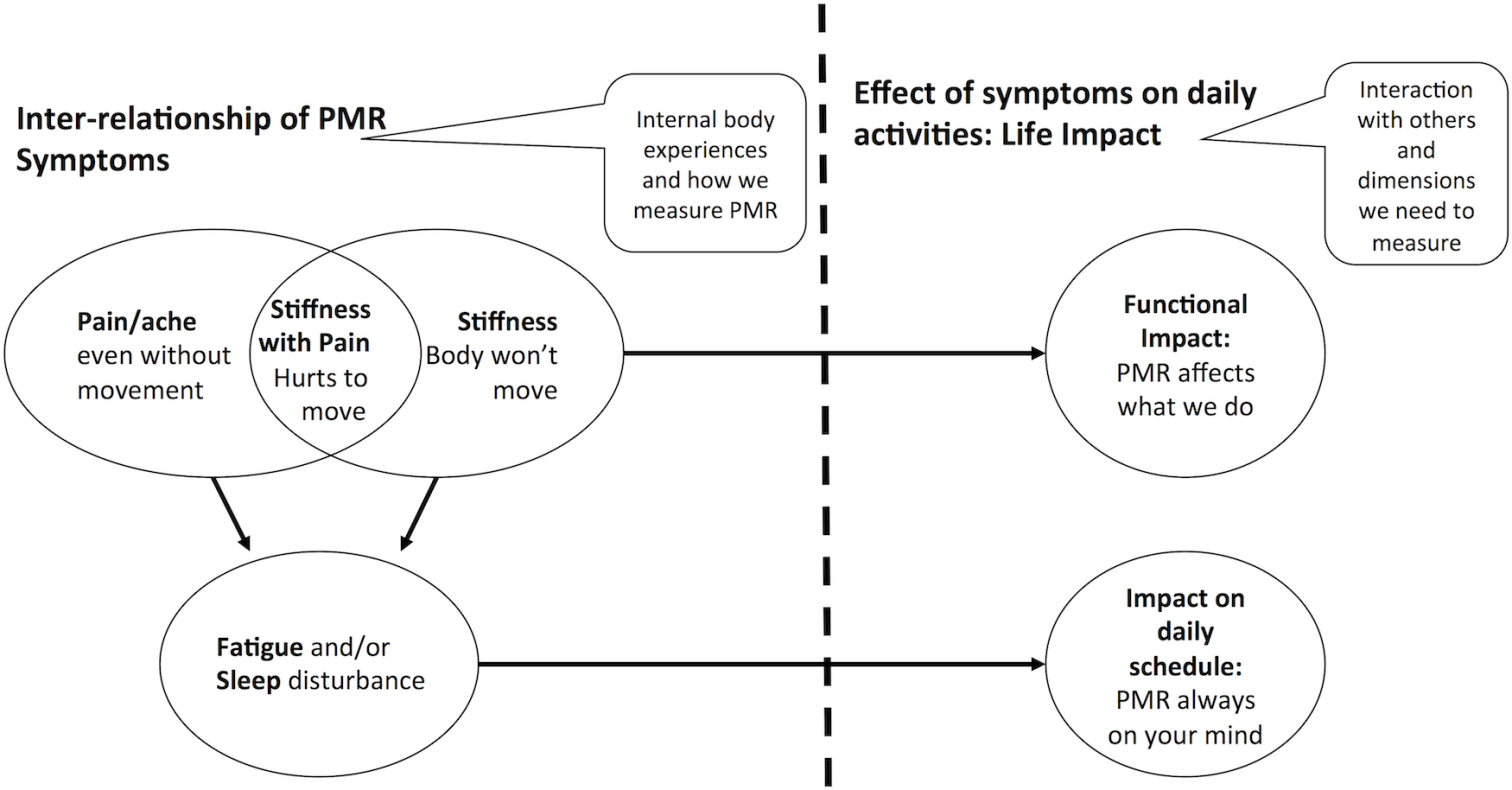
Final conceptual model of the patient experience of stiffness in PMR and aspects that may be relevant to measurement of stiffness. This model reflects the major themes, derived from the data generated by participants with PMR, highlighting the importance of key symptoms and their impact on daily life: limiting function and a constant reminder. Pain and stiffness for many are inextricably linked, with one or other dominating their PMR experience or on occasion difficult to separate out. Outcome measures might address the intensity of symptoms and/or restriction of activities.

These themes interrelate (Fig 1) and are illustrated by exemplar verbatim quotes in Table 3.

**Table 3:** Themes identified during the analysis.

Overarching theme	Theme	Subtheme	Quotations
Body symptoms	Stiffness	“Won’t move”	*“Um I don’t know that you’re conscious of being stiff without moving something*.*”* [SPH/3/M/821-6].
		Unfamiliar experience	*“I’ve never had anything like it ever*.*”* [B/1/M/265-6]
	Making sense of stiffness	Loss of control	“*It’s as if you’ve lost control of—of—of your movement*. *It’s—it’s—you’re wanting to do it like mad*, *but something else is [laughs] stopping you*.*”* [L/1/F/835-41]
		Resistance to glucocorticoids	*I would agree that you have the pain till the steroids kick in and get rid of it*. *And then the stiffness comes afterwards really*. [B/2/F/438]
	Ache/pain: “hurts” (regardless of movement)	Overwhelming pain	“*The pain that I got from PMR was mind-blowing and virtually unique*.” [SPH/2/M/1220-1]
		Ache (a pain that could be ignored)	*“When it was sort of bearable it was just like a dull ache”* [B/1/M/381-2]
	Overlap of stiffness and pain (“hurts to move”)	Stiffness with pain	“*they’re intrinsically linked*, *pain and stiffness*. * *.* *. *So you lift your arm*, *and it’s stiff*, *and then the pain is excruciating*, *that’s how I’d explain it*.*”* [SPH/3/F/176-87]
		Stiffness is pain	*“stiffness is painful*, *the feeling is painful”* [L/3/F/305-6]
	Fatigue (tiredness) and sleep disturbance	Fatigue always present	“*Stiffness is in the background*. *I get up and I straighten up and then it’s forgotten*. *But the um tiredness*, *the fatigue*, *is always present*.” [B/2/M/742-4]
Impact on function: PMR affects what we can do.	“Fundamental” (everyday or valued) activities		“*It’s um hmm*, *a bad day for me would be when I have difficulty moving about*, *getting up from a chair*, *moving about*, *cooking”*. [L/1/M/628-31]
Impact on daily schedule: PMR always on your mind	24-hour cycle of symptom severity	Anticipation	*“But I knew what I was going to have in the night*. *I’d go off to sleep fine*. *But I would wake up 3*.*00 and 4*.*00*, *from then on till midday the next day I would be in agony*.*”* [SPH/3/F/378-89]
Measuring stiffness in PMR	Measure stiffness directly	A 1 to 10 scale	*I would measure it from 1–10*.*”* [SPH/1/F/270-9]*”I wouldn’t like to grade anything on a 1–10 basis personally”* [L/1/F]
		Duration of morning stiffness	*“Um I find that very difficult to quantify*. *Um because it’s not the same every day*.*”* [L/1/M]
	Measure indirect effects of stiffness	Ability to perform everyday activities	*“you could say how difficult or not difficult it was getting up and down the stairs or getting up and out of bed*.” [SPH/1/F/1963-7]

### Stiffness: won’t move

The labels “stiffness” and “pain”, often used interchangeably, were felt to be ways of conveying the *unfamiliar experience* of PMR.

*“I think (the thing) is that the—the—we call it pain or call it stiffness* … *but there are certain things that are indicators that something’s not right*. [SPH/3/F/945-51]

“*I never had anything like it*, *nothing like*. *I’ve never had anything like it ever*.” [B/1/M/265-6]

Stiffness was defined as a restriction of movement, and was a different experience from pain, even if sometimes both were experienced together. Participants kept returning to the idea that it was easiest to describe stiffness in terms of what it stopped them doing, which included many everyday or “fundamental” activities.

“*Er I had a very sore shoulder*, *and that was pain there*. *But then one morning I woke up and I can only say I felt I was in a coffin*, *in that restricted place*, *absolutely solidly stiff*.”[L/2/F/609-11]

“*Yeah well about hard to define it*, *it’s hard to get up out of a chair*, *I know that*.” [B/1/M/1849-50]

Asked to elaborate further, participants explained stiffness using mechanical ideas: for example, a machine that was not working (*loss of control*), or their body moving through a viscous substance or being weighed down by something very heavy (which could in itself be painful). These quotes illustrate the close relationship of the idea of stiffness to the idea of movement.

“*I completely like lost my drives*, *if you like*, *in a machine*, *like my drives*, *complete lack of power*.*”* [B/1/M/724-5]

“*Well I used to think my brain’s [laughs] not telling me how to get out the bath*. *I—I couldn’t push on my arms or anything like that*.*”* [SPH/3/F/90-91]

“*And I’ve tried to analyse whether I’m so stiff because it hurts to go down[stairs] faster*, *or whether it’s like a gloopy glue that you’re trying to work your way through*, *which I find quite strange*.*”* [SPH/2/F/95-98]

“*Stiffness is the worst*. *I’ve got it at the moment*. *And I try to turn round to you*, *it is like I have um bricks on top of each other*, *cemented onto my neck*. *It is really heavy and it is very uncomfortable*.*”* [SPH/2/F/268-71]

### Ache/pain: hurts regardless of movement

Some participants preferred the word ache rather than pain.

*“It wasn’t a pain um I get sometimes in my arms at night now*, *but it’s like a toothache*, *it’s more like an ache*, *you know*.” [SPH/3/F/41-3]

However particularly prior to initiation of glucocorticoid treatment, some participants reported that their pain was *overwhelming* and real, dominating all other symptoms.

“*Pain*, *it’s like when they say about childbirth*, *you’ve forgotten it straight away*, *you know*, *pain you do forget*. *Except I can remember the real extreme*.” [SPH/1/F/932-4]

*“But when I started with the real pain*, *it was 4* ^*th*^ *November*, *you know*, *just at 4 o’clock on the Friday night*.” [L/2/F/127-129]

Pain was often not well-localised to the joints, but they described vivid images of how the pain was felt:

“*I felt that all my muscles had been torn*” [SPH/1/F/477]

“*As if somebody had punched me all over” [SPH/1/F/151-2] “like you’ve been steamrollered by a bus* … *been through a mangle*” [SPH/1/F/3149]

Pain tended to be responsive to medication, whereas responsiveness of stiffness to glucocorticoids varied between individuals:

“*I feel as if the stiffness is the overwhelming symptom*. *I can put up with the pain*. *But it takes over*, *it seems to come from nowhere and take over your body*. *You’ve got—you’ve no sort of—there’s nothing you can actually do*, *is there*? *Whereas you can take a couple of paracetamols and shift the pain*. *But the stiffness was my problem*.” [L/1/F/396-401]

“*Female 1*: *I would agree that you have the pain till the steroids kick in and get rid of it*. *And then the stiffness comes afterwards really*. *Because*, *well*, *you may have had it all along*, *but you don’t realise it because there’s pain*.

*Female 2*: *I haven’t got any stiffness now*. *Do you have stiffness after you’ve had steroids*?

*Female 1*: *Oh yeah I still get stiff*.” [B/2/F’s/438-46]

Participants spent some time discussing whether other conditions could complicate assessment of pain and stiffness; although they were clear that the symptoms prior to treatment were definitely PMR, some participants expressed uncertainty as to whether persistent pain and stiffness symptoms in treated PMR were due to the PMR itself or another cause, such as osteoarthritis.

### Overlap of stiffness and pain

Several patients described pain and stiffness as two separate experiences:

“*Well it’s two different things to me*. *The pain is one thing and the stiffness is another*. *Now if I’m not stiff*, *I’m OK*.” [L/1/F/769-70]

For others, pain and stiffness occurred at the same time, especially when movement produced an increase in pain:

“*stiffness is painful*” [L/3/F/305-6]

For others, the words pain and stiffness were used almost interchangeably, suggesting the two symptoms were *inextricably linked*, or two facets of the same experience, which can make measuring stiffness complicated:

“*I probably just started off with a general sort of ache*, *a stiffness*. *Um because I always used to go to quite a lot of classes*, *sort of aerobics and things*, *and when I came back I wasn’t sort of recovering quite as well*. *You know*, *I always ached a bit when I came back*, *but I was aching a lot more*, *really stiff*. *Um and I just thought*, *“Oh it’s me age*, *I’ll just sort of work through it*.*” But then sort of like three months on*, *like you*, *it was sort of in bed and just—just agony*, *just really stiff*, *I couldn’t sort of get me jumpers on or anything without help*.” [L/1/F/88-97]

### Fatigue and sleep disturbance

Both stiffness and pain contributed to sleep disturbance, with turning over in bed often described as nearly impossible without waking. Sleep disturbance was a prominent feature of PMR for many participants:

“*I think sleep is a major problem*, *yeah I really do*.*”* [SPH/1/M/1575].

*“I might be asleep and just move in my sleep*, *and—and that woke me straight up*.*”* [L/3/F/763-4]

Fatigue, often called “tiredness”, seemed to be pervasive (*always present*) and was seen as more than simply an effect of insufficient sleep; it was described as a significant symptom of PMR with its own diurnal rhythm:

“*Stiffness is in the background*. *I get up and I straighten up and then it’s forgotten*. *But the um tiredness*, *the fatigue*, *is always present*.” [B/2/M/742-4]

“*Now I felt very tired um during the back end of last summer*. *Er I felt*, *from about*, *oh*, *3 o’clock in the afternoon I’d feel really*, *really tired*.” [L/2/F/125-127]

Although there was variation in the severity and diurnal pattern of fatigue, pain and stiffness between individual patients, all reported an impact on physical function.

### PMR affects what we can do

With stiffness present, everyday activities became “*like walking through treacle*” [L/2/F/1236-8]. Some participants saw the most important aspect of PMR as whether or not they could perform common activities of daily living, such as getting up in the morning, dressing and moving around the house, and other valued activities (such as golf, walking the dog, playing badminton, or caring for family members).

For many participants, pain did not stop them doing their valued activities but stiffness did.

“*And it—it was really the impediment of movement that I was er suffering*, *rather than pain in itself*. *In other words*, *I do get quite a lot of—of pain er with it*. *But the pain doesn’t actually stop me doing things*. *The stiffness does*, *it’s a much greater impediment*, *I think*, *to um er—to generally living life*.” [L/1/M/177-181]

“*Sorry—on the days that I can’t get up the stairs say easily*, *I’m not sure whether I’m stiff or I’m in pain*, *to be honest*. *Because I go up on all fours*, *that’s the only way I can get up there*.” [SPH/1/F/579-82].

### Impact on daily schedule: PMR always on your mind

PMR symptoms had a 24-hour cycle of severity; usually all symptoms were worst in “*the small hours*” of the morning or on waking, although for some participants, pain predominated at night and stiffness during the day.

“I *think nights were pain*, *really painful* … *And through the day was more stiffness*.” [B/1/F/1586-90]

“*You dread the morning to come*. *You don’t want to wake up*” [B/1/F/143]

The diurnal variation in symptoms was dramatic, and prompted several participants to reorganise their activities to the afternoon.

*“Now I can guarantee that in a morning I can hardly get up*, *but by 2 o’clock it’s gone*.*”* [L/3/F/44-45]

### Measuring stiffness

When asked what and how they would measure stiffness and pain, some participants suggested a numerical rating scale (such as *“1 to 5”* or “*1 to 10*”) and some preferred words (“*mild*, *moderate or severe*”); no patient suggested visual analogue scales, a common patient outcome measure. However, rating scales were generally felt to need more context than simply a grading of the symptoms, for example assessment of the *ability to perform everyday activities*.

*“It depends on how you would apply the scale”* [L/1/M/1020]

*“If you can’t get out of bed to go to the toilet there’s something seriously wrong with you*, *isn’t there*? *And from that point of view I was 10”* [L/3/F/446-8]

*“And I think descriptive really*, *rather than 0–10*. *Say—say you’re doing it um through the day*, *and the first one you do is in the morning*, *you could say how difficult or not difficult it was getting up and down the stairs or getting up and out of bed*.” [SPH/1/F/1963-7].

*“It’s more related to activity*, *I think*, *as far as I was—I’m concerned*.*”* [L/1/F/ 563–4]

One participant wondered if stiffness could be quantified using a mechanical device:

“*Could you measure functionality of a limb against a—like a spring or something*.*”* [SPH/2/M/533-4])

After prompting from the facilitator, some agreed that duration of morning stiffness was an aspect that could be measured, since this related to the diurnal variation in symptom severity. However this was reflected back more in terms of function rather than using the word stiffness itself.

“*You know*, *you know*, *er it’s just—there’s no um—but I think the sort of morning one would be quite a good one for me*, *how long it takes you to get—get going really*.” [L/1/F/1230-3]

However, duration of morning stiffness was rarely volunteered spontaneously as a suggestion for how stiffness might be measured, possibly due to stiffness being difficult to quantify due to daily variation.

*“Um I find that very difficult to quantify*. *Um because it’s not the same every day*.*”* [L/1/M/1230-1]

Another participant suggested measuring the change in severity of stiffness over the day, rather than duration of stiffness itself:

*“I would measure it*, *in my mind*, *I would measure it from 1–10*, *how you feel when you get up in the morning*, *and grade it in numbers*, *as 10 being the worst*, *and grade it down to 1*, *throughout the day*, *so you know where you are on a level*. *Because during the day you do feel differently*.*”* [SPH/1/F/274-9]

Participants reported that the symptoms in treated PMR were the same as in untreated PMR “*but very much reduced*”, suggesting that it would be valid to measure them in the same way before and after treatment. “*What about measuring it every time you go down a notch on your steroids*? *So then you measure for a week*.” [SPH/1/F/1892-3]

Four major points emerge from these results. First, patients’ experience of stiffness in PMR can be characterised under eight themes. These fall under overarching headings of bodily symptoms, impact on function, impact on daily schedule and measuring stiffness. Second, many patients found it difficult to distinguish between pain and stiffness, and often used the words interchangeably. Others were able to distinguish them and reported that they had different consequences. Third, the measurement of the impact of stiffness might be best undertaken by the assessment of function. Fourth, the consequences of the symptoms of PMR permeated everything to do with patients’ daily routine. A further observation is that it may prove difficult to discover the attribution of symptoms which may persist or recur after successful treatment with glucocorticoids.

## Discussion

This aim of this study was to explore the patient’s concept of “stiffness” in PMR to ensure that it is measured in a way that is relevant to this. The relationship between stiffness and pain appeared more complex than we had anticipated; furthermore many patients reported fatigue as a significant problem in PMR. Stiffness was defined as inability to move; this loss of control of the body had a devastating impact on ability to perform everyday activities, and participants felt that assessing changes in functional ability would be a valid, though indirect, way to assess changes in stiffness itself.

Participants told us that pain/ache responded well to medication, particularly glucocorticoids, but stiffness responded more variably, and fatigue even less well. Indeed, in most previous studies, not all patients with PMR have a complete response to glucocorticoids [2, 6, 17–18]. This illustrates the contrast between the distillation of diagnostic features in the history from the physician’s perspective [1, 19] and the complexity of the patient experience, particularly once established on glucocorticoids. Whether the persistent symptoms reported by many patients were part of the PMR itself or were due to other conditions is a question that could not be answered within this research study, as it would require a full clinical assessment.

Assessment of stiffness does not appear to be straightforward. Rating scales for stiffness itself were suggested by some, but were felt to lack context. Some patients found the difference between pain and stiffness difficult to characterise, but nevertheless it appears important to measure the symptom of stiffness specifically. There was significant diurnal variation in symptom severity, but the worst time of day was not the same for everybody. The most straightforward way to assess the impact of stiffness was felt to be by assessing ability to do everyday activities. In previous studies the Stanford Health Assessment Questionnaire, or the shorter Modified Health Assessment Questionnaire, have been used; function correlates with other measures of disease activity in PMR and is responsive to change [20, 21]. One participant suggested directly measuring limb “functionality” as a way of assessing stiffness, again emphasising the close relationship between stiffness and function.

Morning stiffness duration was felt to be a separate matter to stiffness severity, but duration of morning stiffness was reported to vary from day to day; this raises the question of whether the observed poor test-retest reliability of morning stiffness duration [6] may be due to fluctuation of the symptom being measured, rather than unreliability of the measurement instrument itself. Despite being under the care of rheumatologists, who use morning stiffness duration as part of their construct of disease activity [18], patients still did not suggest stiffness duration as a way of measuring stiffness. This suggests that it may not translate easily into a patient-reported outcome measure. Our findings are indirectly supported by previous work in RA: a cross-sectional study suggested that the description, severity and duration of “morning stiffness” in rheumatoid arthritis was not very different from that reported by patients with “non-inflammatory” joint disease, and that severity scores were associated with disease activity [22]. When stiffness was measured in patients with RA, “morning stiffness” severity and duration were only moderately correlated, and severity was more responsive to change than duration [23]. However, caution should be employed in extrapolating from one disease and patient population to another.

Limitations of this study include the restriction to the UK and those with English as a first language. We did not require fulfilment of classification criteria for PMR, but all patients had to be diagnosed by a rheumatologist. We did not take a quantitative approach, and as for any qualitative study, generalizability outside this setting remains to be established. However, this study sampled for a range of age, disease durations and disability; and recruited from three different hospitals in the UK, thereby accessing a range of disease experiences and care pathways. At two of the centres the focus groups were co-facilitated by rheumatologists (SLM, CTP, RH). The clinicians’ own experience and understanding of PMR would inevitably have influenced the research process, but some clinical input was deemed important for an understanding of the clinical trial context to which these findings would be ultimately applied. At these two centres focus groups were co-facilitated by a non-clinical researcher (MW), and critical self-reflection of facilitators’ prior knowledge, background and assumptions was encouraged. At the third centre, the focus groups were facilitated entirely by rheumatology nurse researchers trained in qualitative methods. Facilitators were all aware of the importance of focus group methodology whereby the inquiry should come to surround the participants’ agenda and a breadth of opinion is achieved [9]. Analysis was conducted independently by an experienced qualitative researcher (MM), a non-clinical research assistant (MW) and one clinician (SLM). During the project MM provided MW and SLM with informal training in reflexive analytical skills. In addition, a key strength of this work is that patients were involved in data interpretation and preparation for publication [11]: two patients (JD, MN) analysed two transcripts and met with the analysts to ensure that the interpretation of the themes were accurate from their perspective.

Basing the study around a genuine problem (How do we measure stiffness in PMR?) helped to focus the research question, and we used theoretical concepts in outcomes research to develop our conceptual model. This conceptual model, based on the eight themes identified during the analysis, will inform the future development of a core outcome set in PMR.
